# FAK inhibition delays liver repair after acetaminophen-induced acute liver injury by suppressing hepatocyte proliferation and macrophage recruitment

**DOI:** 10.1097/HC9.0000000000000531

**Published:** 2024-10-17

**Authors:** Qing Li, Qi Xu, Jialin Shi, Wei Dong, Junfei Jin, Chong Zhang

**Affiliations:** 1 Guangxi Key Laboratory of Molecular Medicine in Liver Injury and Repair, Affiliated Hospital of Guilin Medical University, Guilin, Guangxi, China; 2 Guangxi Health Commission Key Laboratory of Basic Research in Sphingolipid Metabolism Related Diseases, Affiliated Hospital of Guilin Medical University, Guilin, Guangxi, China; 3 Laboratory of Hepatobiliary and Pancreatic Surgery, Affiliated Hospital of Guilin Medical University, Guilin, Guangxi, China; 4 China-USA Lipids in Health and Disease Research Center, Guilin Medical University, Guilin, Guangxi, China; 5 Department of Hepatobiliary and Pancreatic Surgery, the First Affiliated Hospital of Zhengzhou University, Zhengzhou, Henan, China

## Abstract

**Background:**

Overdose of acetaminophen (APAP), a commonly used antipyretic analgesic, can lead to severe liver injury and failure. Current treatments are only effective in the early stages of APAP-induced acute liver injury (ALI). Therefore, a detailed examination of the mechanisms involved in liver repair following APAP-induced ALI could provide valuable insights for clinical interventions.

**Methods:**

4D-label-free proteomics analysis was used to identify dysregulated proteins in the liver of APAP-treated mice. RNA-Seq, hematoxylin-eosin staining, immunohistochemical staining, immunofluorescence staining, quantitative PCR, western blotting, transwell were used to explore the underlying mechanisms.

**Results:**

Utilizing high throughput 4D-label-free proteomics analysis, we observed a notable increase in proteins related to the “focal adhesion” pathway in the livers of APAP-treated mice. Inhibiting focal adhesion kinase (FAK) activation with a specific inhibitor, 1,2,4,5-Benzenetetraamine tetrahydrochloride (also called Y15), resulted in reduced macrophage numbers, delayed necrotic cell clearance, and inhibited liver cell proliferation in the necrotic regions of APAP-treated mice. RNA-Seq analysis demonstrated that Y15 downregulated genes associated with “cell cycle” and “phagosome” pathways in the livers of APAP-treated mice. Furthermore, blocking extracellular matrix (ECM)-integrin activation with a competitive peptide inhibitor, Gly-Arg-Gly-Asp-Ser (GRGDS), suppressed FAK activation and liver cell proliferation without affecting macrophage recruitment to necrotic areas. Mechanistically, ECM-induced FAK activation upregulated growth-promoting cell cycle genes, leading to hepatocyte proliferation, while CCL2 enhanced FAK activation and subsequent macrophage recruitment via F-actin rearrangement.

**Conclusions:**

Overall, these findings underscore the pivotal role of FAK activation in liver repair post-APAP overdose by promoting liver cell proliferation and macrophage recruitment.

## INTRODUCTION

Acetaminophen (APAP) is one of the most commonly used antipyretic analgesic, but APAP poisoning is also a leading cause of DILI and liver failure.^1^ APAP-induced acute liver injury (ALI) is mainly divided into the initial injury phase and the subsequent repair phase.^2^,^3^ Hitherto, the mechanisms of APAP-induced hepatocyte damage have been studied extensively.^2^,^4^,^5^,^6^,^7^ After entering hepatocytes, APAP is rapidly metabolized into the active product N-acetyl-p-benzoquinonimine, which is conjugated and cleared by intracellular glutathione. When excessive N-acetyl-p-benzoquinonimine cannot be conjugated and cleared by glutathione, it will form adducts with intracellular proteins, thus leading to mitochondrial damage, reactive oxygen species production, c-Jun N-terminal kinase activation, opening mitochondrial permeability transition pores, releasing endonucleases that cause DNA damage, and ultimately causing hepatocyte necrosis.^2^ Additionally, recent studies have shown that ferroptosis, a form of regulated cell death, also plays a role in APAP-induced liver injury.^8^ Unfortunately, N-acetyl cysteine (NAC), which restrains APAP-induced hepatocyte cell death, is only beneficial for patients with APAP poisoning within 8 hours after ingestion.^1^

Subsequently, the liver initiates a series of complex repair processes, including clearing necrotic cells and initiating liver cell proliferation.^2^,^9^,^10^ Necrotic liver cells are mainly cleared by granulocytes and macrophages, and C-C motif ligand 2 (CCL2) mediates macrophage chemotaxis toward the necrotic area through the C-C motif chemokine receptor 2 (CCR2) pathway.^11^,^12^ Epidermal growth factor recepto, hepatocyte growth factor receptor, β-catenin, NF-κB pathway is activated in APAP-induced liver injury and is involved in the proliferation of liver cells.^6^,^13^,^14^ Interestingly, TGF-β and IL-11 were also increased in APAP-induced liver injury while inhibited the proliferation of liver cells.^15^,^16^ Notably, blocking of TGF-β and IL-11 accelerated liver repair and improved survival of mice treated with a lethal dose of APAP. However, there is no literature that comprehensively explores new mechanisms or pathways that regulate liver repair after APAP-induced liver injury at the proteomic level.

Focal adhesions are composed of extracellular matrix (ECM), membrane receptors (integrins), focal adhesion kinase (FAK), and intracellular skeleton proteins, and play important roles in embryonic development, immune response, morphogenesis, and wound healing.^17^ After binding to the ECM, integrins recruit FAK, promote its aggregation, and autophosphorylation, thereby activating downstream signaling pathways.^18^ Notably, FAK plays a central role in the signal transduction of the focal adhesion pathway. In addition, cytokines such as HGF and EGF can also activate FAK.^19^,^20^,^21^ At the cellular level, FAK activation promotes cell survival, proliferation, adhesion, and migration in different cell types.^22^ Interestingly, deletion of FAK in hepatocytes accelerates liver proliferation after 2/3 partial hepatectomy in mice.^23^ Hitherto, the role of FAK in modulating liver repair after APAP-induced liver injury remains unexplored.

Based on 4D-label-free proteomics analysis, we found that proteins enriched in “focal adhesion” pathway were upregulated in the liver of APAP-treated mice. Moreover, blocking FAK activation by 1,2,4,5-Benzenetetraamine tetrahydrochloride (Y15) not only decreased macrophage recruitment to the necrotic area, delayed the clearance of necrotic cells, but also inhibited proliferation of liver cells, thus delaying liver repair after APAP-induced ALI. Mechanistically, ECM activates FAK and promotes the initiation of cell cycle in liver cells; CCL2 recruits macrophage to the necrotic area in a p-FAK-dependent manner. Our study suggests that enhancing FAK activation may serve as a novel therapy for APAP-induced ALI.

## METHODS

### Cell line

Mouse hepatocyte cell line alpha mouse liver 12 was cultured in DMEM/F-12(1:1) medium containing 5% fetal bovine serum (FBS, Gibco) and 40 ng/mL dexamethasone (MCE, Cat. #HY-14648). Mouse macrophage cell line RAW264.7 was purchased from Guangzhou Cellcook Biotech Co., Ldt (Guangzhou, China) and cultured in DMEM medium containing 10% FBS. All cells were cultured at 37 °C with 5% CO_2_.

### ALI model and drug intervention

C57BL/6J mice were purchased from Hunan Slyke Jingda Laboratory Animal Co. Ltd (Hunan, China) and fed in the specific pathogen-free house. After water-only fasting for 15 hours, male mice 6 to 7 weeks of age were intraperitoneally injected with 300 mg/kg of APAP (MCE, Cat. #HY-66005) to induce the ALI model. In parallel, the mice injected with equivolumetric saline solution were used as controls. For KC depletion, mice were injected with clodronate liposome (Yeasen, Cat. 40337ES08) 3 days before APAP treatment. For blocking FAK activation, ALI mice were intraperitoneally injected with 30 mg/kg of Y15 (MCE, Cat. #HY-12444) at 10 and 26 hours after APAP exposure. For blocking ECM-mediated integrin activation, ALI mice were intraperitoneally injected with 240 mg/kg of Gly-Arg-Gly-Asp-Ser (GRGDS) (GL Biochem, China, 151842) or GRGES (GL Biochem, 052654) at 10 and 26 hours after APAP exposure. The mice were harvested at indicated time points. Serums were collected for detecting the levels of ALT and AST, and liver tissues were collected for western blotting, hematoxylin-eosin (H&E) staining and immunohistochemical (IHC) experiments. For survival rate of APAP-treated mice, a lethal dose of APAP (500 mg/kg) was used. All experiments involving animals were carried out in accordance with the guidelines set forth in the Guide for the Care and Use of Laboratory Animals (NIH Publication No. 80-23, revised 1996) and the ARRIVE (Animals in Research: Reporting In Vivo Experiments) and received approval from the ethical standards established by Guilin Medical College.

### H&E, IHC, and double immunofluorescence staining

Fresh samples of mice liver were collected, embedded in paraffin, and cut into slices. Tissue slices were dewaxed and then used for subsequent experiments. Liver tissue pathomorphology was measured by H&E staining. Hepatocyte cell death was detected by terminal deoxynucleotidyl transferase (TdT) dUTP Nick-End Labeling assay staining assay using the Servicebio Fluorescein Tunel Cell Apoptosis Detection Kit (Servicebio, China), according to the instructions. For IHC, antigen retrieval was performed by microwave heating in EDTA buffer (pH 9.0) for FAK (1:250, abcam, ab40794), CD68 (1:1000, abcam, ab283654), Ly6G (1:1000, abcam, ab238132), and S100A9 (1:1000, abcam, ab242945) or in citrate buffer (10 mM, pH 6.0) for Ki67 (1:800, CST, 12202). After incubation with primary and secondary antibodies, slices were stained using the chromogenic substrate diaminobenzidine (CST, 8059S) and restained with hematoxylin. Five random fields of images were recorded under the microscope (DFC 7000T, LEICA).

Double immunofluorescence staining was performed using the double-fluorescence IHC mouse/rabbit kit (Immunoway, Suzhou, China) with anti-hepatocyte nuclear factor 4 alpha (1:2000, abcam, ab201460) or anti-CD68 (1:300, abcam) and anti-FAK (1:250, abcam, ab40794) primary antibodies. Immunofluorescent images were photographed using a laser confocal microscope (AX+N-STORM, Nikon).

### Proteomic and RNA-Seq analysis

To identify the differentially expressed proteins (DEPs) that were involved in the repair phase of APAP-induced ALI, total proteins extacted from 3 individual liver tissues of saline-treated or APAP-treated mice were applied to 4D-label-free quantitative proteomics by Zhongke Xinsheng Biotechnology Co., Ltd (Zhejiang, China). To identify the differentially expressed genes (DEGs) that were affected by Y15, total RNAs extracted from 3 individual liver tissues of the saline-treated, APAP-treated, and APAP+Y15–treated mice were applied to RNA-Seq by Tsingke Biotechnology Co., Ltd. (Tianjin, China).

Rstudio was used for bioinformatics analysis. The R Package “DEseq2” was used to construct a differential expression analysis dataset. The criterion of |log_2_FC| >1 and false discovery rate<0.05 was used as a threshold to select DEGs or DEPs. Hierarchical cluster of DEPs and DEGs was presented by heatmap using the R Package “pheatmap.” Volcano plot showing the DEPs was conducted by using the R Package “ggplot2.” The R Package “clusterProfiler” was used to conduct kyoto encyclopedia of genes and genomes (KEGG) pathways enrichment analysis of DEPs and DEGs.^24^

### Analysis of mRNA expression by qPCR

Total RNAs was isolated using RNA isolater Total RNA Extraction Reagent (R401-01, Vazyme, China). Reverse transcription of total RNAs was performed by the ToloScript All-in-One RT EasyMix (TOLOBIO, Shanghai, China). Then, the cDNAs were used to detect the mRNA levels of target genes using the 2×Q3 SYBR quantitative polymerase chain reaction Master mix Kit (TOLOBIO). The sequences of primers are shown in Supplemental Table S1, http://links.lww.com/HC9/B42.

### Western blotting

Proteins extracted from cells or tissues were denatured, and then separated on 4%–20% polyacrylamide gels and subsequently transferred to PVDF membrane (Millipore, MA). After blocking with 5% skim milk powder for 1 hour, the membrane was separately incubated with anti-pFAK (1:10000, abcam, ab76244) or anti-FAK (1:2000, abcam, ab40794) at 4 °C overnight and then incubated with secondary antibodies at room temperature for 1 hour. After washing off the unbound antibodies by Tris-buffered saline plus Tween-20 buffer, the membrane was exposed using enhanced chemiluminescence substrate (ABclonal, China) and photographed by Tanon 5200 Chemiluminescence Imaging System (Tanon, Shanghai, China).

### Transwell assay

RAW264.7 cells (1.0×10^5^) suspended in 100 μL serum-free medium with or without 10 μM Y15 or 150 μg/mL GRGDS were seeded in the transwell (5 μm, Corning) and then put into the 24-well plates containing serum-free medium with or without 20 ng/mL CCL2. After 12 hours, all transwells were taken out and fixed with methanol, and stained with crystal violet. Five random fields of transmigrated macrophages in each well were photographed and counted.

### F-actin staining

Twenty-four hours after seeding, RAW264.7 cells were washed with PBS twice, fixed with 3.7% formaldehyde, permeabilized with 0.1% Triton X-100, stained with Actin-Tracker Red (1:100, Beyotime, China, C2203S) followed by DAPI staining according to manufacturer’s instruction, and then photographed using a laser confocal microscope.

### Statistical analysis

Gray levels of protein expression in each lane were calculated by Image-J software. All data were presented the means±SEM using GraphPad Prism software (version 9.0). Unless otherwise mentioned, paired and unpaired *t* test were conducted to analyze the differences between the 2 groups. The *p*-value<0.05 was considered significant. *, *p*<0.05; **, *p*<0.01; ***, *p*<0.001; ****, *p*<0.0001; ns, not significant.

## RESULTS

### Proteins enriched in “focal adhesion” pathway are increased in APAP-induced liver injury

**FIGURE 1:**
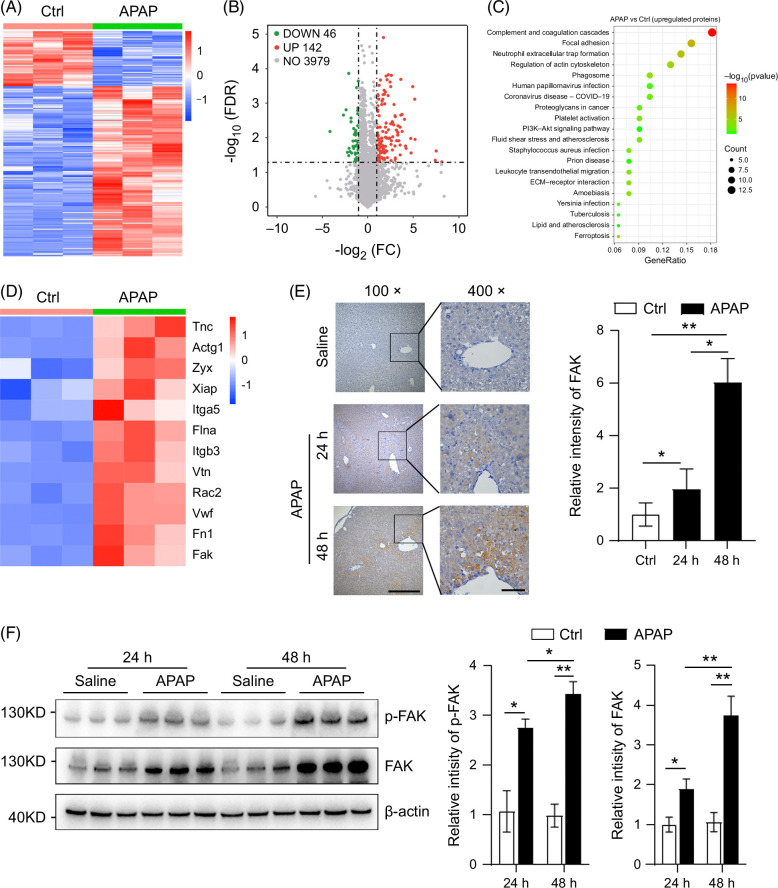
Proteins enriched in “focal adhesion” are increased in APAP-induced liver injury. (A, B) Heatmap and volcano diagram showing the differentially expressed proteins in the liver between Ctrl and APAP-treated mice. (C) KEGG analysis of upregulated proteins in the liver of APAP-treated mice compared with saline-treated mice. (D) Heatmap showing upregulated proteins involved in the “focal adhesion” pathway. (E) Expression of FAK was increased in the liver of APAP-treated mice as shown by immunohistochemical staining (left) and the relative intensity of FAK was presented (right). n=4/group. Scale bar, 50 μm. (F) Protein expression of FAK and p-FAK was increased in the liver of APAP-treated mice, as demonstrated by western blotting (left), and their relative intensity is presented (right). *, *p*<0.05; **, *p*<0.01; ***, *p*<0.001. Abbreviations: APAP, acetaminophen; Ctrl, control; ECM, extracellular matrix; FAK, focal adhesion kinase; FC, fold change; FDR, false discovery rate.

To screen out the key proteins that participated in the repair phase of APAP-induced liver injury, 4D-Lable-free proteomics was used to detect the protein level in the liver of control mice (Ctrl) and mice treated with APAP for 48 hours. By bioinformatic analysis, a total of 188 DEPs, including 142 upregulated and 46 downregulated proteins, were selected (Figure 1A, B and Supplemental Dataset 1, http://links.lww.com/HC9/B43). KEGG enrichment analysis of upregulated proteins revealed “complement and coagulation cascades” and “focal adhesion” as the 2 highest-ranked KEGG pathways (Figure 1C). The roles of complement and coagulation cascades in APAP-induced liver injury have been reported previously.^9^,^10^,^25^ Therefore, we focused on the potential function of focal adhesion in APAP-induced liver injury. Upregulated proteins in the focal adhesion pathway included FAK, a tyrosine kinase which plays a central role in integrin-mediated signal transduction (Figure 1D). IHC and H&E staining showed that FAK expression gradually increased in the necrotic region of APAP-treated mice (Figure 1E and Supplemental Figure S1, http://links.lww.com/HC9/B42). More importantly, western blotting showed that total and phosphorylated FAK were also increased in the liver of APAP-treated mice (Figure 1F). These results indicate that FAK is activated in APAP-induced ALI and may be involved in the process of liver repair.

### Blocking FAK activation inhibits liver repair after APAP administration

**FIGURE 2:**
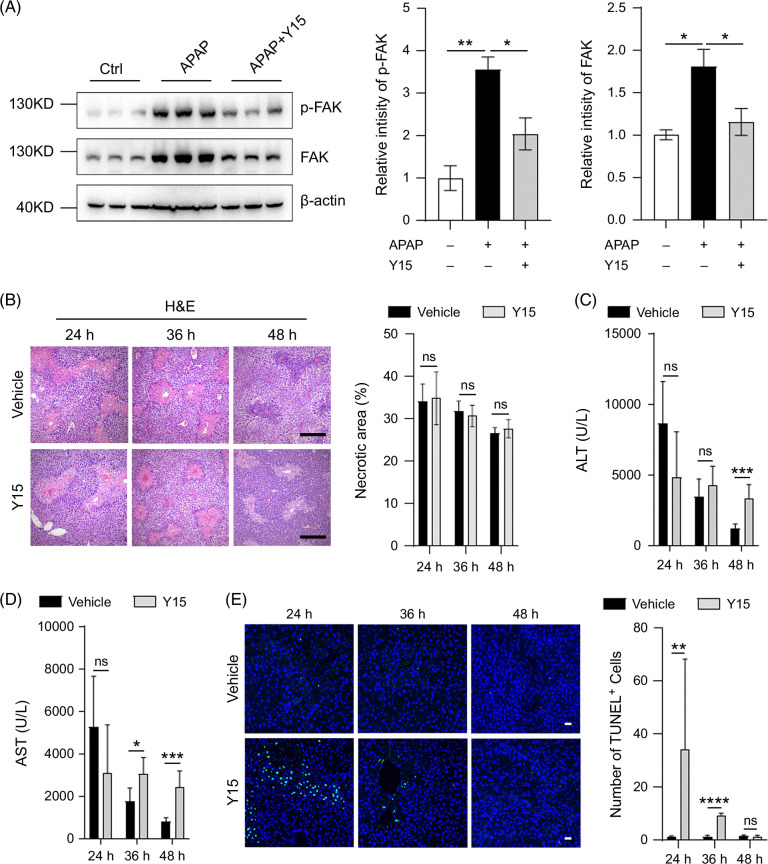
Blocking FAK activation inhibits liver repair after APAP administration. (A) Protein expression of p-FAK and FAK were suppressed by Y15 in the liver of mice treated with APAP for 36 hours as shown by western blotting and their relative intensity was presented. n=3/group. (B) Y15 had no effect on necrotic area of liver in APAP-treated mice as shown by H&E staining (left) and the necrotic area was shown (right). Scale bar, 250 μm. n=5–6/group. (C, D) The effect of Y15 on serum ALT and AST levels in APAP-treated mice. n=5–7/group. (E) Y15 increased TUNEL-positive cells in the liver of APAP-treated mice. Representative images are displayed on the left, with the number of TUNEL-positive cells presented on the right. Scale bar, 50 μm. n=5–6/group. *, *p*<0.05; **, *p*<0.01; ***, *p*<0.001; ****, *p*<0.0001. Abbreviations: APAP, acetaminophen; Ctrl, control; FAK, focal adhesion kinase; H&E, hematoxylin-eosin; TUNEL, terminal deoxynucleotidyl transferase (TdT) dUTP Nick-End Labeling assay.

To evaluate the role of FAK during the process of APAP-induced ALI, Y15, a specific small-molecule inhibitor of FAK, was used to block the function of FAK. It is worth noting that Y15 was given 10 and 26 hours after APAP administration, so Y15 was not able to affect the initial injury phase of APAP-induced ALI, including N-acetyl-p-benzoquinonimine formation, glutathione depletion, and c-Jun N-terminal kinase activation, which occurs within 9 hours after APAP treatment in mice.^26^ As shown by western blotting, Y15 restrained APAP-induced increase of total and phosphorylated FAK in the liver (Figure 2A). Interestingly, Y15 did not impact the necrotic area in the liver of APAP-treated mice (Figure 2B). However, Y15 did not alter serum ALT and AST levels in the early phase of APAP-induced ALI, but notably increased these levels in the late phase (Figure 2C, D). Furthermore, Y15 did not affect the number of terminal deoxynucleotidyl transferase (TdT) dUTP Nick-End Labeling assay-positive cells in the liver 14 hours post-APAP treatment (Supplemental Figure S2, http://links.lww.com/HC9/B42) but did increase this number at 24 and 36 hours post-treatment (Figure 2E), indicating that Y15 did not influence the initial injury phase post-APAP exposure. Given that cell death occurs before the release of ALT and AST, and the clearance of these enzymes in the serum takes time, it is postulated that Y15 delays the elimination of necrotic cells in the livers of APAP-treated mice. Moreover, Y15 significantly reduced the survival rate of mice when exposed to a lethal dose of APAP (Supplemental Figure S3, http://links.lww.com/HC9/B42). These results suggest that FAK activation may play a critical role in liver repair following APAP-induced ALI.

### Blocking FAK activation inhibits hepatocyte proliferation and macrophage recruitment

**FIGURE 3:**
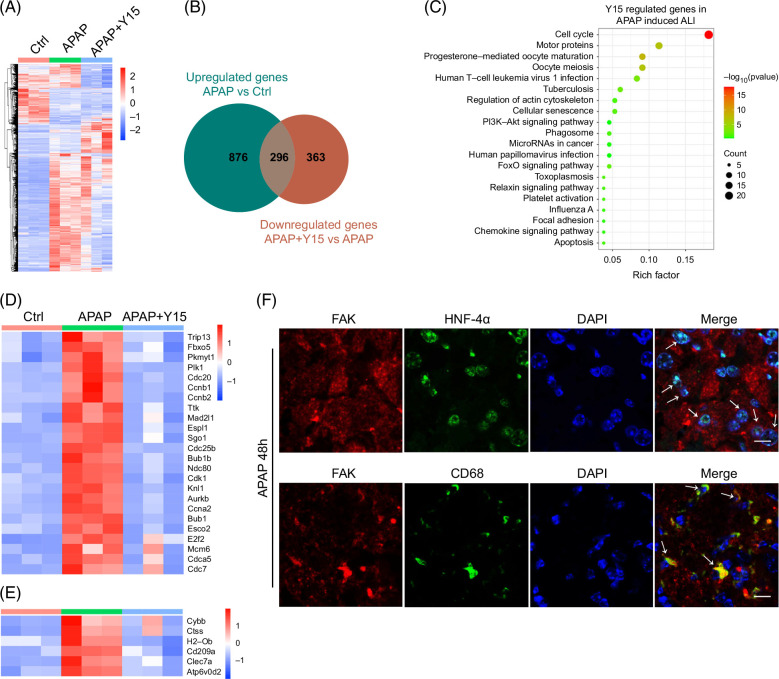
Blocking FAK activation inhibits “cell cycle” and “phagosome” pathways in the liver of APAP-treated mice. (A) Heatmap revealing the differentially expressed genes in the liver of saline-treated (Ctrl), APAP-treated and APAP+Y15–treated mice. Mice were harvested 48 hours after APAP administration. (B) Venn diagram showing the number of exclusive and common genes between upregulated genes in the liver of APAP-treated mice verssus saline-treated mice and downregulated genes in the liver of APAP+Y15–treated mice versus APAP-treat mice. (C) KEGG analysis of Y15-regulated genes in the liver of APAP-induced ALI. (D, E) Heatmap showing the upregulated genes involved in “cell cycle” (D) and “phagosome” (E) pathways. (F) Immunofluorescence staining showed the colocalization of FAK (red) and HNF-4α (green) or CD68 (green) in the liver after APAP treatment for 48 hours. Scale bar, 10 μm. For (A–E), n=3/group. Abbreviations: ALI, acute liver injury; APAP, acetaminophen; Ctrl, control; FAK, focal adhesion kinase; HNF-4α, hepatocyte nuclear factor 4 alpha.

**FIGURE 4:**
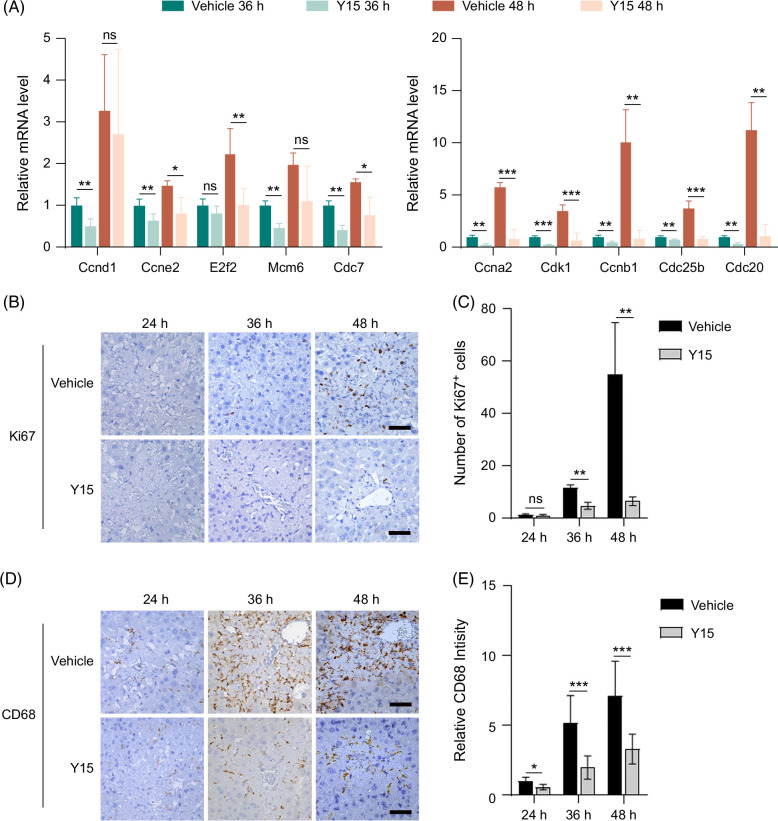
Blocking focal adhesion kinase activation inhibits liver cell proliferation and macrophage recruitment in the liver of acetaminophen-treated mice. (A) Y15 decreased growth-promoting cell cycle genes in the liver of acetaminophen-treated mice as shown by qPCR analysis. n=4–6/group. (B–E) Y15 decreased the number of Ki67+ (B) and CD68+ (D) cells in the liver of acetaminophen-treated mice as shown by immunohistochemical staining, and the number of Ki67+ cells (C) and relative intensity of CD68 (E) were presented. Scale bar, 50 μm. n=5–6/group. *, *p*<0.05; **, *p*<0.01; ***, *p*<0.001.

To explore the underlying mechanism by which FAK activation mediated liver repair after APAP administration, we performed RNA-Seq to identify dysregulated genes in the liver between Ctrl-treated and APAP-treated mice or APAP-treated and APAP+Y15–treated mice (Figure 3A and Supplemental Dataset 2, http://links.lww.com/HC9/B44). Logically, FAK-induced genes were those that were upregulated in the liver of APAP-treated mice (APAP vs. Ctrl) but decreased in the liver of APAP+Y15–treated mice (APAP+Y15 vs. APAP) (Figure 3B). Luckily, 296 genes were sorted out and then applied to KEGG enrichment analysis (Figure 3B). Among the enriched pathways, “cell cycle” and “phagosome,” which are involved in the proliferation of liver cells and removal of necrotic cells, respectively, are involved in the process of liver repair (Figure 3C–E). Moreover, double immunofluorescence staining revealed that FAK was expressed on hepatocyte nuclear factor 4 alpha–positive or CD68-positive cells (Figure 3F), indicating that hepatocytes and macrophages in the liver of APAP-treated mice expressed FAK. Next, we validated the RNA-Seq results by quantitative polymerase chain reaction. Indeed, Y15 decreased growth-promoting cell cycle genes in the liver of mice 36 or 48 hours after APAP treatment (Figure 4A). Moreover, IHC staining showed that Y15 reduced the number of Ki67^+^ cells and CD68^+^ macrophages in the liver of APAP-treated mice (Figure 4B–E). To distinguish peripheral blood monocyte–derived macrophages and KCs, KCs were depleted using a single injection of clodronate liposome (Supplemental Figure S4A, http://links.lww.com/HC9/B42). Consistently, Y15 also decreased the number of CD68^+^ cells in the liver of KC-depleted mice following APAP exposure (Supplemental Figure S4B, http://links.lww.com/HC9/B42). However, Y15 had no effect on the recruitment of neutrophils (Ly6G^+^) and S100A9^+^ inflammatory cells to the liver of APAP-treated mice (Supplemental Figure S5, http://links.lww.com/HC9/B42). IHC and H&E staining indicated that the increased macrophages were predominantly located in the necrotic areas of APAP-treated mice (Supplemental Figure S6, http://links.lww.com/HC9/B42). Taken together, these findings suggest that FAK activation may play a crucial role in liver cell proliferation and the recruitment of peripheral blood monocyte–derived macrophages to the necrotic areas of APAP-treated mice, thereby facilitating liver repair post-APAP-induced injury.

### Blocking integrin activation inhibits liver cell proliferation without affecting macrophage recruitment

**FIGURE 5:**
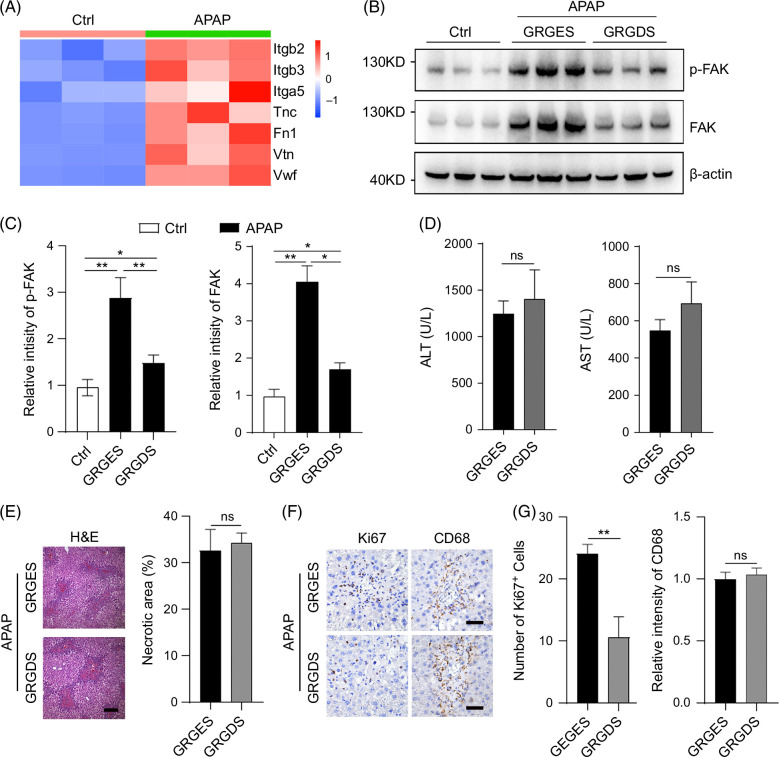
Blocking extracellular matrix/integrin-mediated FAK activation inhibits liver cell proliferation without affecting macrophage recruitment in APAP-induced acute liver injury. (A) Heatmap revealing increased expression of extracellular matrixs and integrins in the liver of APAP-treated mice compared with saline-treated mice. n=3/group. (B, C) GRGDS decreased p-FAK and FAK level in the liver of APAP-treated mice. Mice were harvested 36 hours after APAP treatment. n=3/group. (D) GRGDS had no effect on serum ALT and AST levels in APAP-treated mice. n=4/group. (E) GRGDS had no effect on the necrotic area in APAP-treated mice. Scale bar, 250 μm. n=3/group. (F, G) GRGDS inhibited liver cell proliferation but not macrophage recruitment in the liver of APAP-treated mice. Representative images of Ki67^+^ and CD68+ cells (F). The number of Ki67^+^ cells (G, left) and relative intensity of CD68 (G, right) per field were presented. Scale bar, 50 μm. n=3/group. For (D–G), mice were harvested 48 hours after APAP treatment. *, *p*<0.05, **, *p*<0.01. Abbreviations: APAP, acetaminophen; Ctrl, control; FAK, focal adhesion kinase; H&E, hematoxylin-eosin; GRGDS, Gly-Arg-Gly-Asp-Ser; GRGES, Gly-Arg-Gly-Glu-Ser.

ECM-integrin ligation is the most important way to activate FAK and plays a vital role in disease progression. Notably, several ECMs and integrins were dramatically increased in the liver of APAP-treated mice (Figure 5A). Therefore, a competitive peptide inhibitor, GRGDS, was used to block the interaction between ECMs and integrins.^27^,^28^ As expected, GRGDS decreased the level of p-FAK and FAK in the liver of APAP-treated mice (Figure 5B, C). However, GRGDS had no effect on serum ALT, AST levels, and necrotic area in the liver of APAP-treated mice (Figure 5D, E). Interestingly, GRGDS decreased the number of Ki67^+^ cells while did not affect the number of CD68^+^ cells in the liver of APAP-treated mice (Figure 5F, G). These results suggest that ECM/integrins-mediated FAK activation promotes the proliferation of liver cells without affecting macrophage recruitment after APAP-induced liver injury.

### ECM-integrins-FAK axis plays a vital role in hepatocyte proliferation in vitro

**FIGURE 6:**
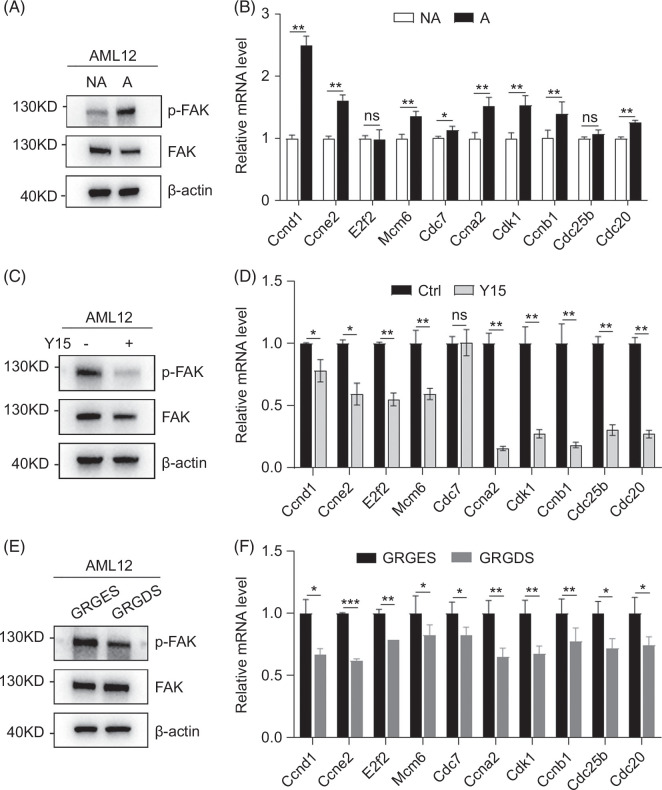
ECM-integrins-FAK axis plays a vital role in hepatocyte proliferation in vitro. (A, B) Adhesive cells had higher p-FAK level and increased expression of growth-promoting cell cycle genes. One hour after seeding in ultralow attachment plates (NA) or TC-treated plates (A), protein expression of FAK and p-FAK were detected by western blotting. Twelve hours after seeding in ultralow attachment plates (NA) or TC-treated plates (A), the mRNA of indicated genes were detected by qPCR. (C, D) Y15 decreased p-FAK level and expression of growth-promoting cell cycle genes in adhesive AML-12 cells. (E, F) GRGDS decreased p-FAK level and expression of growth-promoting cell cycle genes in adhesive AML-12 cells. For (C–F), proteins and RNAs of AML-12 cells treated with vehicle or Y15 (10 μM) or GRGES (150 μg/mL) or GRGDS (150 μg/mL) were harvested 1 and 12 hours, respectively, after seeding in TC-treated plates. *, *p*<0.05, **, *p*<0.01, ***, *p*<0.001. Abbreviations: AML, alpha mouse liver; FAK, focal adhesion kinase; GRGDS, Gly-Arg-Gly-Asp-Ser; GRGES, Gly-Arg-Gly-Glu-Ser.

To further demonstrate the role of ECM/integrins/FAK axis in mediating hepatocyte proliferation, we detected growth-promoting cell cycle genes under different conditions in vitro. Cells easily attach to tissue culture-treated plates (adhesive cells) while remain suspended in ultralow attachment plates (nonadhesive cells). Compared with nonadhesive cells, adhesive cells had elevated p-FAK levels and increased expression of several growth-promoting cell cycle genes in alpha mouse liver 12 cells, a murine hepatocyte cell line (Figure 6A, B). In addition, both Y15 and GRGDS decreased p-FAK levels and growth-promoting cell cycle genes in adhesive alpha mouse liver 12 cells (Figure 6C–F). These results suggest that ECM/integrin-mediated FAK activation is required for hepatocyte proliferation.

### Different roles of integrin and FAK activation in macrophage recruitment in vitro

**FIGURE 7:**
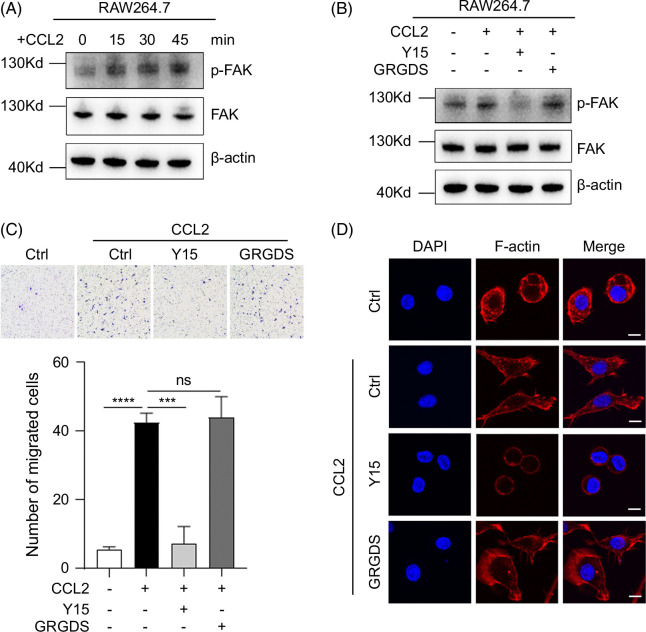
CCL2-mediated macrophage recruitment depends on FAK activation. (A) CCL2 increased p-FAK level in RAW264.7 cells. RAW264.7 cells were stimulated with the CCL2 (20 ng/mL) for indicated time points. (B) The effect of Y15 and GRGDS on CCL2-induced FAK activation in RAW264.7 cells. RAW264.7cells were stimulated with or without CCL2 (20 ng/mL) supplemented with Y15 (10 μM) or GRGDS (150 μg/mL) as indicated for 20 minutes. (C) The effect of Y15 and GRGDS on CCL2-directed migration of RAW264.7 cells. (D) The effect of Y15 and GRGDS on CCL2-induced F-actin rearrangement in RAW264.7 cells. Nuclei were stained by DAPI. Scale bar, 10 μm. ***, *p*<0.001, ****, *p*<0.0001. Abbreviations: CCL2, C-C motif ligand 2; Ctrl, control; FAK, focal adhesion kinase; GRGDS, Gly-Arg-Gly-Asp-Ser.

As shown above, Y15 but not GRGDS impaired macrophage recruitment to necrotic areas in the liver of APAP-treated mice, so we explored the underlying mechanism in vitro. CCL2 is a well-known chemokine that recruits macrophages to injured tissues. Interestingly, CCL2 increased p-FAK levels in RAW264.7 cells, a mouse macrophage cell line (Figure 7A). Moreover, CCL2-induced FAK activation could be blocked by Y15, but not by GRGDS (Figure 7B). Consistently, CCL2-directed macrophage migration was inhibited by Y15, but not by GRGDS (Figure 7C). F-actin rearrangement is required for cell movement during chemotaxis, therefore, we explored whether Y15 and GRGDS affected CCL2-induced F-actin rearrangement. As expected, CCL2 induced a dramatic F-actin rearrangement, morphology change and increased pseudopods formation in RAW264.7 cells, which was blocked by Y15, but not by GRGDS (Figure 7D). These results suggest that CCL2-induced chemotaxis of macrophages is dependent on FAK activation while ECM-integrin ligation may not be involved in CCL2-induced chemotaxis of macrophages.

Collectively, these data demonstrate that FAK activation is required for liver repair after APAP-induced liver injury and suggest enhancing FAK activation as a potential therapy for APAP overdose.

## DISCUSSION

**FIGURE 8:**
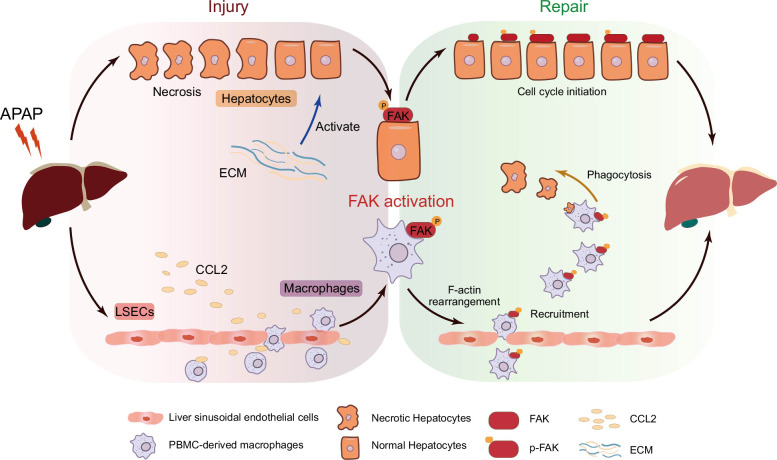
Graphic summary of the role of FAK activation in promoting liver repair after APAP-induced acute liver injury. ECM and CCL2 levels are elevated in the liver of mice following APAP treatment. On one hand, FAK is activated by ECM, which promotes cell cycle initiation and the proliferation of liver cells. On the other hand, FAK is also activated by CCL2, and this activation is essential for the CCL2-mediated chemotaxis of PBMC-derived macrophages to the necrotic areas of the liver in APAP-treated mice, thereby facilitating the removal of necrotic cells and debris through phagocytosis. Collectively, these processes enhance liver repair following APAP-induced acute liver injury. Abbreviations: APAP, acetaminophen; CCL2, C-C motif ligand 2; FAK, focal adhesion kinase; LSECs, liver sinusoidal endothelial cells; PBMC, peripheral blood monocyte.

In this article, we found that FAK was activated following APAP-induced ALI. Increased ECM/integrin-activated FAK and promoted the proliferation of liver cells. Additionally, FAK activation was required for CCL2-induced recruitment of macrophages to the necrotic area (Figure 8). Our study discovers a key role of FAK activation in promoting liver repair after APAP overdose and suggests modulating FAK activation as a potential therapy for APAP overdose.

The molecular mechanisms by which APAP overdose causes hepatocyte cell death have been explored extensively.^2^ Based on these findings, NAC, which prevents hepatocyte cell death, becomes the mainstay of therapy for patients with APAP overdose. However, NAC is only effective when given within 8 hours for humans after ingestion of excessive APAP and 4 hours for mice.^29^,^30^ Moreover, prolonged NAC treatment even delayed liver repair due to decreased proliferation of liver cells.^31^ Encouragingly, several studies demonstrated the existence of molecules that exacerbated liver injury and delayed liver repair after ALI and blocking or removal of these molecules efficiently mitigated liver injury. Liver regeneration was inhibited in mice treated with a high dose of APAP compared to those with low dose of APAP.^13^ Larsen et al^32^ reported that high-volume plasma exchange significantly enhanced the outcome of transplantation-free patients with acute liver failure. Inhibition of TGF-β signaling promoted the regeneration of hepatocytes, thus promoting survival of mice given a lethal dose of APAP.^15^ When administered at 6 and 24 hours after APAP ingestion, engineered FGF19, which increased the proliferation of hepatocytes, was more efficient than NAC in reducing the mortality of mice given a lethal dose of APAP.^33^ Even when administered 10 hours after APAP ingestion, a neutralizing IL11RA antibody significantly improved the survival of mice treated with a lethal dose of APAP by promoting the regeneration of liver cells.^16^ These studies strongly suggest that enhancing the proliferation of liver cells is a promising strategy to treat patients with APAP poisoning.

In our study, we found that FAK was increased and activated in the liver of APAP-treated mice, and blocking FAK activation significantly decreased the proliferation of liver cells. Even though the role of FAK in promoting cell proliferation has been well studied, we found that FAK activation was required for the regeneration of liver cells following APAP exposure for the first time. Blocking FAK activation decreased growth-promoting cell cycle genes, including Ccnd1, Ccne2, etc., both in vitro and in vivo, suggesting FAK activation is required for cell cycle initiation during liver repair. Increased ECM expression was at least partially responsible for FAK activation and proliferation of liver cells since blocking ECM-integrin interaction by GRGDS decreased p-FAK level, expression of growth-promoting cell cycle genes, and number of Ki67^+^ cells in vitro or in vivo. In addition, we found that Y15 was more efficient than GRGDS in decreasing Ki67^+^ cells in liver of APAP-treated mice. This is reasonable because of the following facts. First, EGF and HGF can promote hepatocyte proliferation after injury,^2^,^34^ and their cognate receptors epidermal growth factor recepto and hepatocyte growth factor receptor are activated in the liver following APAP exposure in mice.^13^ Second, plasma HGF level is increased in patients with APAP overdose.^35^ Third, although ECMs are primary ligands for integrins that activate FAK,^22^ EGF and HGF can also activate FAK through their cognate receptors to some degree.^36^,^37^ Therefore, we speculate that ECM, together with EGF and FGF, promote proliferation of liver cells following APAP poisoning in a p-FAK-dependent manner. Recently, Matchett et al^38^ uncovered a novel ANXA2+ migratory hepatocyte subpopulation that mediates wound closure following APAP-induced liver regeneration. They also demonstrated that wound closure occurs prior to hepatocyte proliferation, indicating an uncoupling of these 2 processes. Furthermore, FAK activation plays a role in mediating cell migration;^39^ additional experiments will be necessary to further investigate its role in facilitating wound closure after APAP-induced liver regeneration.

Removal of dead cells is necessary for liver repair after injury.^2^ Macrophages are the primary phagocytes that are recruited to the necrotic area and participate in debris removal and liver regeneration following liver injury.^11^,^12^ Moreover, macrophages may also promote the proliferation of liver cells by secreting HGF, IL-6, and TNF-α.^40^,^41^ Herein, we observed a strong negative correlation between the number of macrophages and dead cells. This was evidenced by increased TUNEL^+^ positive cells and a decreased number of CD68^+^ macrophages in APAP+Y15 mice compared with APAP mice at the repair phase. It is well known that CCL2-CCR2 signaling drives the recruitment of macrophages to necrotic areas or inflamed tissues.^42^ The molecular mechanisms of chemotaxis mediated by chemokines and its cognate receptors have been extensively studied. Briefly, the binding of chemokines to chemokine receptors activates and dissociates heterotrimeric G-proteins (Gαβγ), thus activating downstream PI3K and Rho family of small GTPases, which leads to actin polymerization and pseudopod formation.^43^,^44^ Herein, we found that CCL2 activated FAK and induced F-actin rearrangement and pseudopods formation, which could be blocked by Y15 but not GRGDS, suggesting FAK activation is required for CCL2/CCR2-mediated chemotaxis. However, how FAK is activated by CCL2/CCR2 needs further investigation.

Taken together, we found a vital role of FAK in mediating liver repair after APAP-induced ALI. FAK is activated by ECM and promotes the proliferation of liver cells. Additionally, FAK is activated by CCL2 and its activation is required for CCL2-mediated chemotaxis of macrophages to necrotic area of the liver in APAP-treated mice.

## Supplementary Material

## Data Availability

All data supporting the findings of this study are available within the paper and its supplementary information. The raw proteomic datasets (Supplemental Dataset 1, http://links.lww.com/HC9/B43) and raw RNA-Seq datasets (Supplemental Dataset 2, http://links.lww.com/HC9/B44) in this study can be found in online repository. The names of the repository and accession number(s) can be found at https://doi.org/10.6084/m9.figshare.25634505. Chong Zhang conceived the project. Junfei Jin and Wei Dong supervised the project. Qing Li, Qi Xu, and Jialin Shi performed the experiments and analyzed the data. Chong Zhang and Junfei Jin provided the fund support. Chong Zhang and Qing Li wrote the manuscript with editing input from all authors. This study was supported in part by Special Project Guangxi Science and Technology Base and Talent (GUIKE AD20238031), the Natural Science Foundation of Guangxi (2020GXNSFDA238006), Guangxi Natural Science Foundation of China (2024GXNSFAA010040), the Special Fund of the Central Government Guiding Local Scientific and Technological Development by Guangxi Science and Technology Department (GuikeZY21195024), National Natural Science Foundation of China (82260499), the third batch of Lijiang Scholar Award in Guilin (2022-5-07), the Science and Technology Planned Project in Guilin (20210102-1), the Medical High Level Talents Training Plan in Guangxi (G202002005), the Construction Fund of Guangxi Health Commission Key Laboratory of Basic Research in Sphingolipid Metabolism Related Diseases (the Affiliated Hospital of Guilin Medical University, ZJC2020005), and grant from MOE Key Laboratory of Gene Function and Regulation. The authors have no conflicts to report.
