# Supplementary material for: FAK inhibition delays liver repair after acetaminophen-induced acute liver injury by suppressing hepatocyte proliferation and macrophage recruitment
Source: Hepatol Commun. 2024 Oct 17;8(11):e0531. doi: 10.1097/HC9.0000000000000531 (PMC11495758; doi:10.1097/HC9.0000000000000531)
Supplement: Supplementary file 1 [file hc9-8-e0531-s001.doc]

**
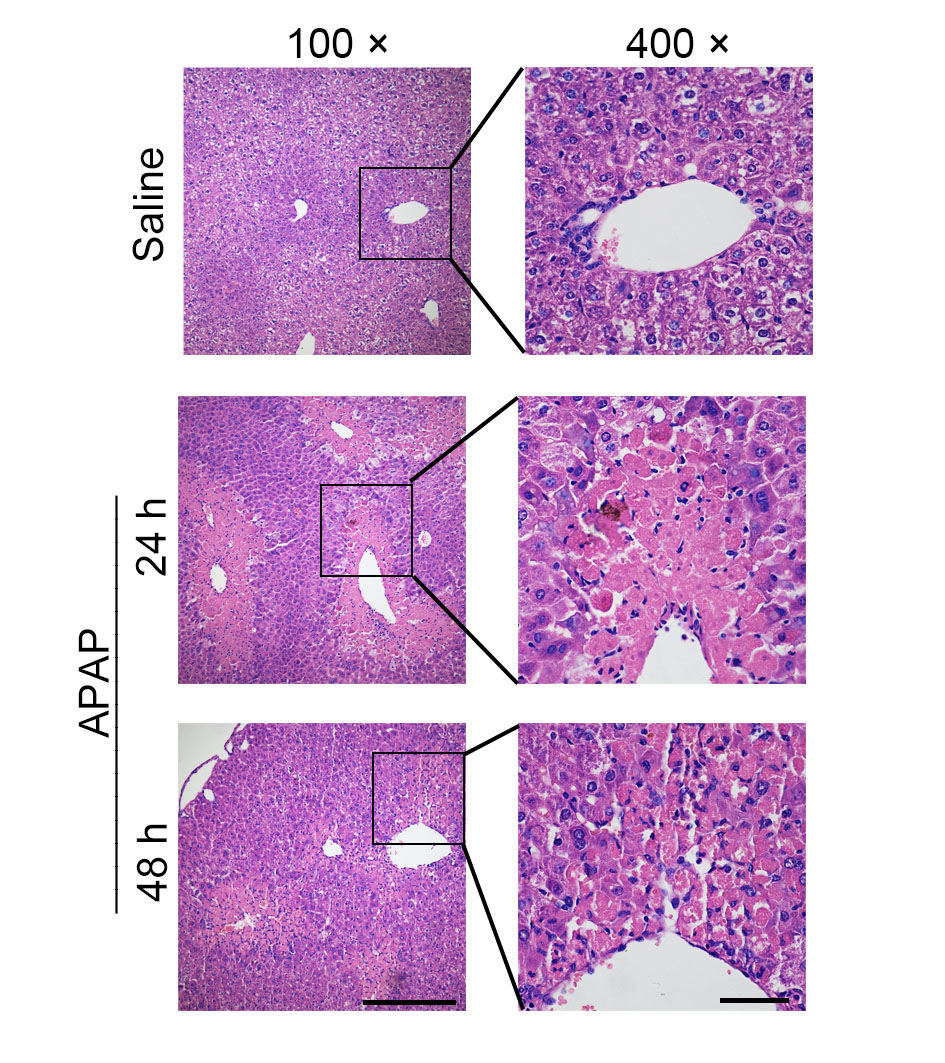
**

**Supplementary Figure 1. H&E staining of the liver at different time points following APAP treatment.** H&E staining was performed with sections adjacent to those used for IHC in Figure 1E. Scale bar, 250 μm (left panel), 50 μm (right panel).


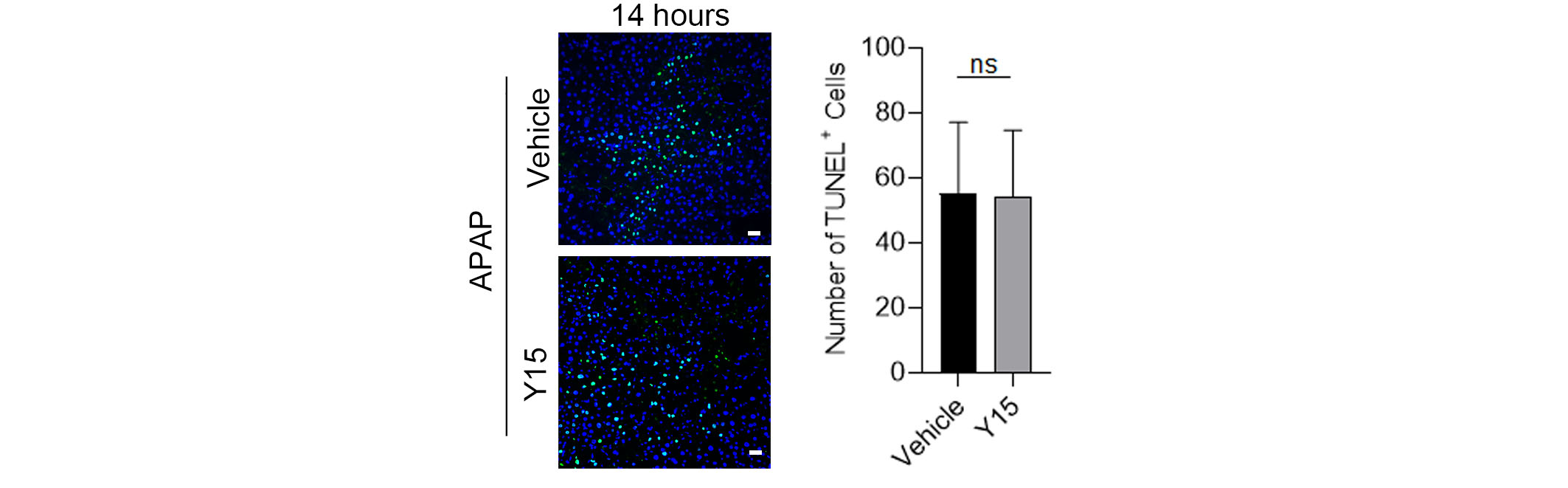


**Supplementary Figure 2. Y15 did not affect the number of TUNEL positive cells in the liver 14 hours post APAP treatment.** Fourteen hours after APAP treatment, mice form vehicle- or Y15-treated mice were harvested and liver tissues were subjected to TUNEL staining. Representative images are displayed on the left, with the number of TUNEL positive cells presented on the right. Scale bar, 50 μm.

**
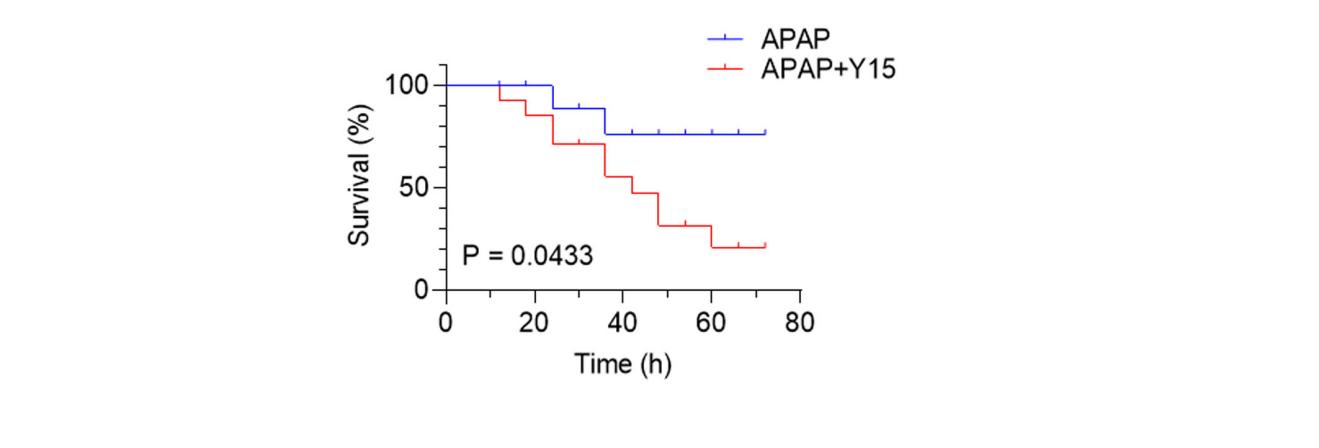
**

**Supplementary Figure 3. Y15 decreased survival rate of APAP-treated mice.** Mice were given a lethal dose of APAP (500 mg/kg), followed by vehicle (n = 10) or Y15 (n = 10) administration 10 hours after APAP treatment.


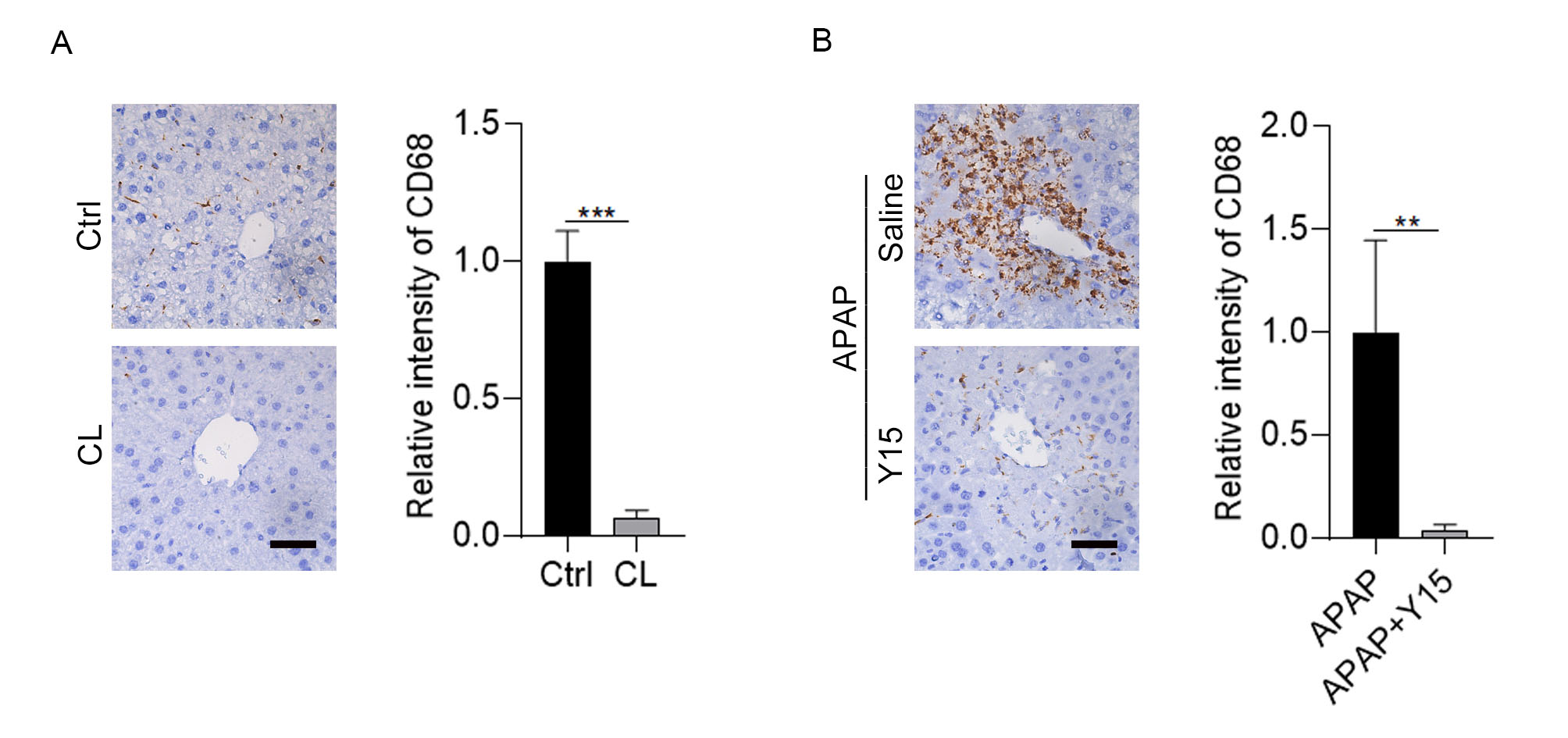


**Supplementary Figure 4. Y15 decreased the number of CD68^+^ cells in the liver of Kupffer cell-depleted mice after APAP exposure.** (A) Clodronate liposome (CL) depleted Kupffer cells in the liver. Four days after a single injection of CL, mice were harvested and liver tissues were subjected to IHC staining for CD68. (B) Three days after CL injection, mice were fasted, treated with APAP and saline or Y15 as described in the Materials and Methods. Forty-eight hours after APAP treatment, liver tissues were harvested and subjected to IHC staining for CD68. Representative images are displayed on the left, with the relative intensity of CD68 presented on the right. Scale bar, 50 μm.

**
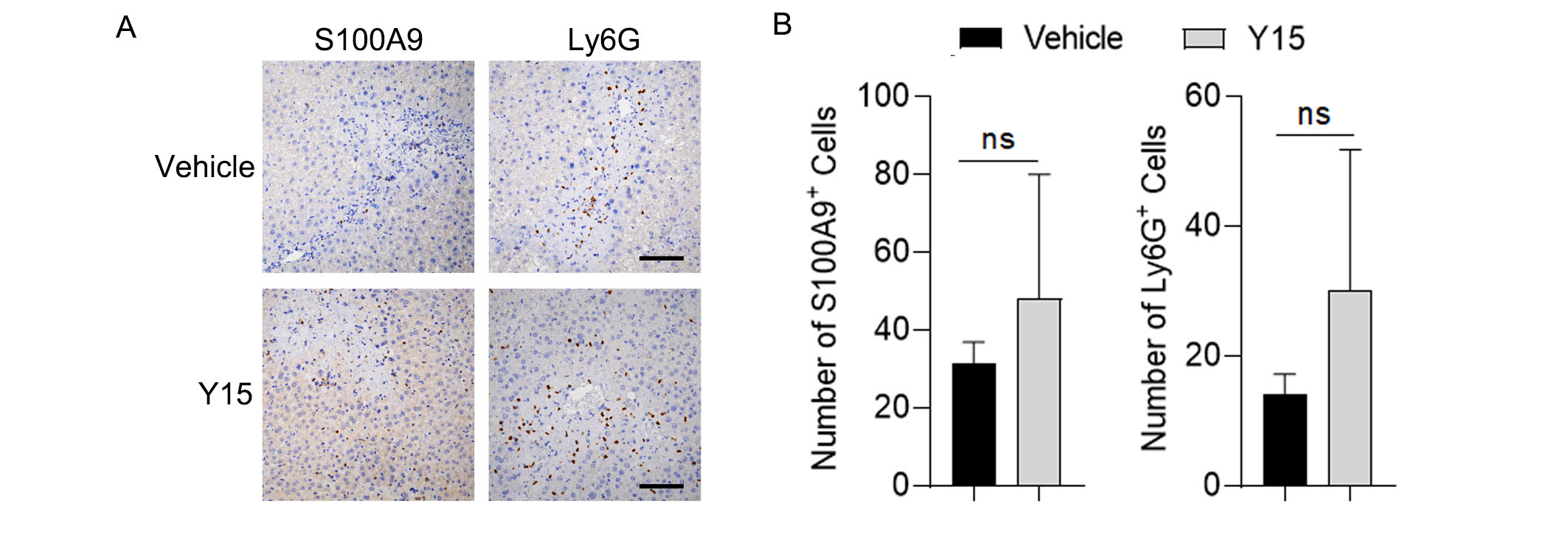
**

**Supplementary Figure 5. Y15 had no effect on the recruitment of S100A9^+^ and Ly6G^+^ cells in liver of APAP-treated mice.** (**A, B**) S100A9^+^ and Ly6G^+^ cells were detected by IHC (A) and their numbers per filed were presented (B). Mice were harvested 48 hours after APAP treatment. Scale bar, 50 μm. Ns, not significant.


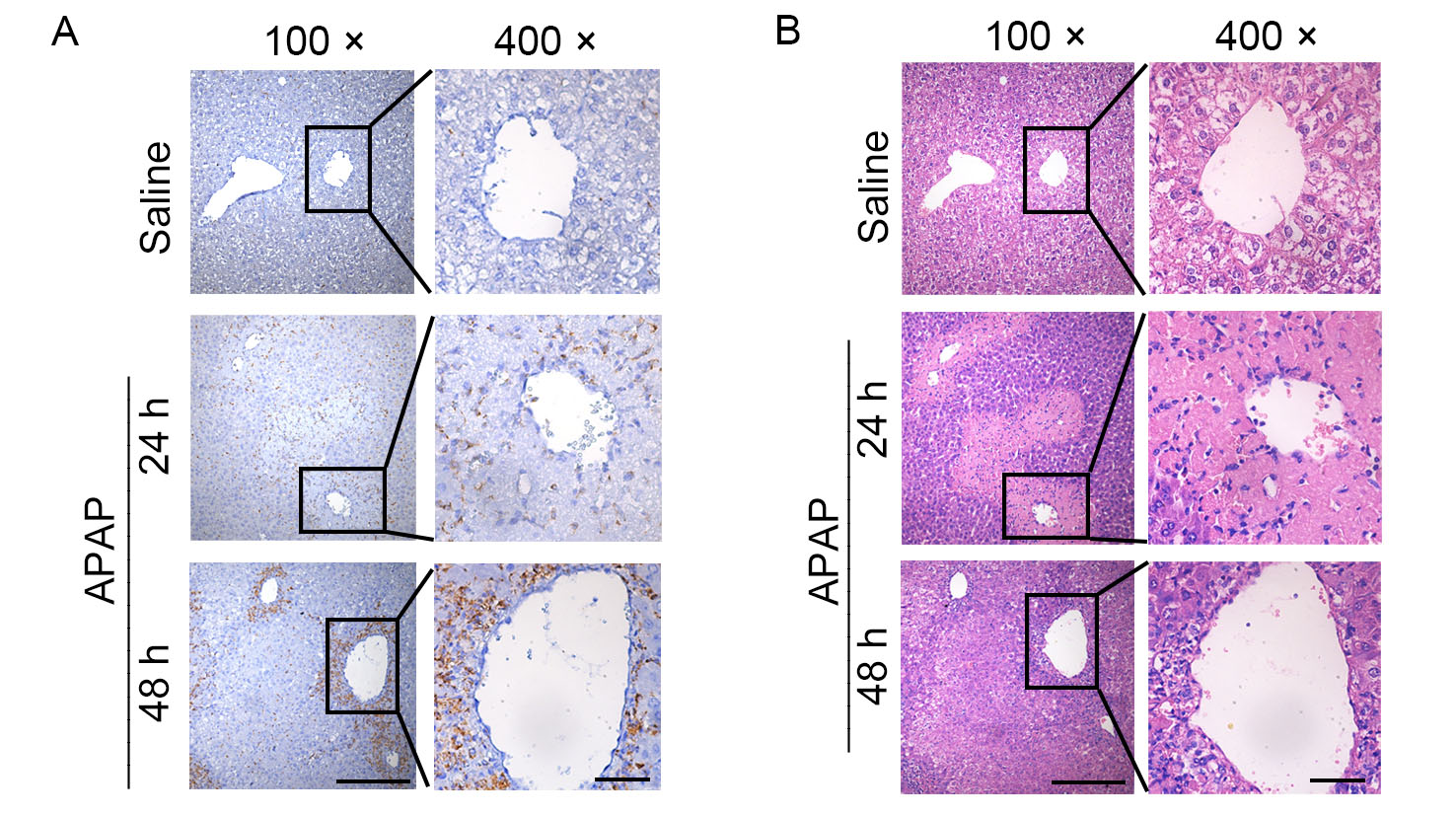


**Supplementary Figure 6.** Macrophages infiltrated the necrotic area of APAP-treated mice. After 24 and 48 hours of APAP treatment, mouse livers were harvested and subjected to IHC staining for CD68 (A) or H&E (B) staining. Two adjacent sections were used for IHC staining and H&E staining, respectively. Scale bar, 250 μm (left panel), 50 μm (right panel).

| **Supplementary Table 1. Primers for RT-PCR** | | |
| --- | --- | --- |
| **Name** | **Sense strand/Sense primer (5'-3')** | **Antisense strand/Antisense primer (5'-3')** |
| **Primers for RT-PCR** | | |
| ACTIN | CCCTGAAGTACCCCATTGAA | CTTTTCACGGTTGGCCTTAG |
| CCNA2 | GCCTTCACCATTCATGTGGAT | TTGCTCCGGGTAAAGAGACAG |
| CCNB1 | GCGTGTGCCTGTGACAGTTA | CCTAGCGTTTTTGCTTCCCTT |
| CCNB2  CCND1 | AGCTCCCAAGGATCGTCCTC  CGTACCCTGACACCAATCTC | TGTCCTCGTTATCTATGTCCTCG  ACTTGAAGTAAGATACGGAGGGC |
| CCND2 | GAGTGGGAACTGGTAGTGTTG | CGCACAGAGCGATGAAGGT |
| CCNE2 | AGCCGTTTACAAGCTAAGCAA | TGGCCTGAATTATCTGGGTTTC |
| CDC7  CDC20  CDC25B  E2F2  MCM6 | AACAGCGTGATGAGGGAAACT  GTTCGTGTTCGAGAGCGATTT  GCCACCTCTCGGTCTTTGAG  ACGGCGCAACCTACAAAGAG  ACCAACCCAAGGTTTGGAGG | CGCTCTGAATCCTGGTGTG  CTAGGGGTGGTCTGAACCTT  TGGTCTGTGTAAGAGTGGTAACC  GTCTGCGTGTAAAGCGAAGT  TAATGCTCTCAGCGGTCTGTT |
